# The New Microbiology: an international lecture course on the island of Spetses

**DOI:** 10.1093/femsml/uqac026

**Published:** 2023-03-31

**Authors:** Pascale Cossart, Roberto Kolter, Bruno Lemaitre, Athanasios Typas

**Affiliations:** Pascale Cossart, Institut Pasteur, 28 rue du docteur Roux, Paris, 75015, France; European Molecular Biology Laboratory ( EMBL) Meyerhofstrasse 1, Heidelberg, Germany; Harvard Medical School, 25 Shattuck street, Boston Massachussetts 02115, United States; EPFL, 1015 Lausanne, Switzerland; European Molecular Biology Laboratory ( EMBL) Meyerhofstrasse 1, Heidelberg, Germany

**Keywords:** Bacterial Gene Regulation, Infection Biology, Antibiotic Resistance, Symbiosis, Phage Defense, Viromes

## Abstract

In September 2022, an international summer course entitled ‘The new microbiology’ took place in Greece, on the island of Spetses. The organizers aimed to highlight the spectacular advances and the renaissance occurring in Microbiology, driven by developments in genomics, proteomics, imaging techniques, and bioinformatics. Combinations of these advances allow for single cell analyses, rapid and relatively inexpensive metagenomic and transcriptomic data analyses and comparisons, visualization of previously unsuspected mechanisms, and large-scale studies. A ‘New Microbiology’ is emerging which allows studies that address the critical roles of microbes in health and disease, in humans, animals, and the environment. The concept of one health is now transforming microbiology. The goal of the course was to discuss all these topics with members of the new generation of microbiologists all of whom were highly motivated and fully receptive.

## Introduction

Spetses Summer courses started in 1966 focused on molecular biology and were led and attended by many of the ‘giants’ of the emerging field. By the time the courses celebrated their 50^th^ anniversary they had diversified to cover many different areas of the life sciences. The original course was started and continued by Marianne Grunberg-Manago with a molecular biology focus and offered every four years. In 1998, two of the present course organizers (Pascale Cossart (PC) and Roberto Kolter (RK)) were invited to lecture in this course. At the conclusion of that course, Marianne Grunberg-Manago asked PC to take over the organization of the course, which she did with RK since 2002.

Microbiology has witnessed a real renaissance in the last 30 years. The landscape has changed tremendously since the ‘golden era’ of *E. coli* genetics and molecular biology of the 1960s and 1970s. Some sub-disciplines of microbiology are evolving at an impressively increasing speed, particularly those based on genomic and post genomic approaches. Techniques have changed. Microbiologists must also be ‘sequencers,’ ‘cell biologists,’ ‘physiologists,’ and ‘immunologists.’ Needless to say, microbiology laboratories are in a better position when they are able to integrate numerous approaches. The whole field has evolved and boundaries with other disciplines (cell biology, ecology, bioinformatics, physiology, immunology) are vanishing.

Nearly three decades ago, the field of infection biology began exploding when classical genetics, molecular biology, and cell biology merged, giving rise to Cellular Microbiology. This theme was the focus of the Spetses course that we (PC and RK) organized in 2002. New aspects of Microbiology have now emerged. The study of microbial communities in the environment and in plant and animal hosts is now in spectacular expansion, highlighting their importance in ecology, and in human health and disease. In the environment, microbial communities are critical to ecological and biogeochemical processes such as decomposition, plant growth, and nutrient cycling. Appreciation of the composition of the host-associated microbiotas (bacteria, fungi, viruses) and their important roles in human health has grown enormously. The intestinal microbiota plays a key role in host nutrition, metabolism, immune cells homeostasis, and in gut-brain interactions. Dysbiosis, corresponding to qualitative, quantitative, and functional changes in the intestinal microbiota, in response to stresses (e.g. antibiotic treatments) has been associated with diseases such as inflammatory bowel disease, metabolic syndrome and more recently neurologic and psychiatric disorders. Approaches such as personalized diets, use of probiotics or fecal transplantation, aiming at modifying the composition and/or the activity of the intestinal microbiota, represent hopeful strategies to treat dysbioses. Much research is still required to help define, and maybe engineer, a ‘healthy’ microbiota. Microbiomes and symbioses are more and more accessible to systematic studies. Results from these studies indicate that these symbioses are not only critical for human health, but also for plant health. In fact, virtually every ecosystem depends on microbial communities for its function. The concept of ‘one health’ needs to be well understood and considered by young scientists. In addition, fundamental microbiology has also recently made spectacular progresses demonstrating the critical role of RNA in many regulatory processes, including in defense against phages as first demonstrated by the discoveries of CRISPR systems.

**Figure 1.**
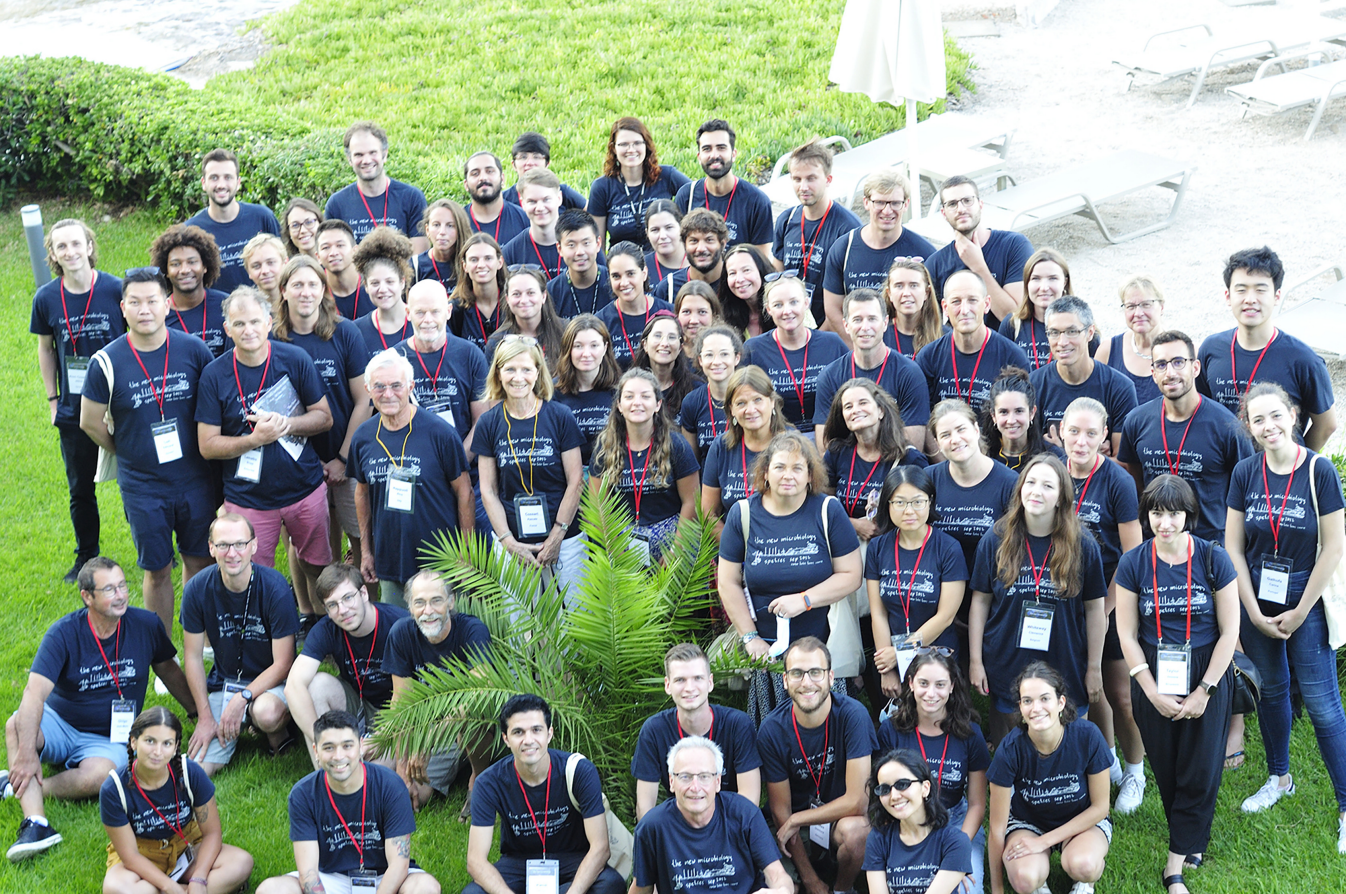
Group Photo.

The 2022 course (https://meetings.embo.org/event/22-new-microbiology) covered microbiomes and symbioses, RNA regulation, phages and phage defense systems, as well as antibiotic resistance, infections and some innate immunity mechanisms. The 2022 course, organized by the four authors of this article, was a tremendous success due to the strong commitment of the 23 lecturers and the unreserved enthusiasm of the 50 students (Fig. 1). Probably the fact that it was one of the first such events after the pandemic also contributed to stimulate interest!

## General topics

**Pascale Cossart** (Institut Pasteur, Paris, France & EMBL, Heidelberg, Germany) opened the course by giving an historical perspective on the evolution of microbiology from the discovery of ‘animalcules’ by A. van Leeuwenhoek (1632–1723) up to the present New Microbiology. She highlighted the discoveries of Louis Pasteur and Robert Koch at the end of the nineteenth century, with the first human vaccinations, followed by the discoveries of plant and animal viruses, of bacteriophages by Felix d'Hérelle in 1917, and that of penicillin in 1929. She showed how bacteria and phages contributed to the establishment of the role of DNA as the genetic material and later to the development of molecular biology and genetic engineering. DNA sequencing allowed gigantic progresses in three areas of microbiology: infection biology, fundamental microbiology, and socio-microbiology. She also described how the combined use of molecular biology, cell biology, microscopy, genomics, and bioinformatics led to a revolution in infection biology with ‘Cellular Microbiology’ emerging in the late eighties (Cossart et al. 1996, Cossart 2005). She then discussed that transcriptomics revealed the many regulatory roles played by RNA, in particular small RNAs with the unexpected discovery of CRISPRs and their use in genome editing (Cossart 2018). More recently, metagenomics and data analyses make it possible to address the critical role in health and disease, of various microbiotas in humans, animals, plants, and the environment. Cossart ended her talk by introducing the ‘One health,' concept which was further discussed during the course (see below).

**Roberto Kolter** (Harvard Medical School, Boston, USA), in the second lecture of the course, discussed the general topic of microbial diversity in the context of some general principles of microbial ecology. After discussing the gargantuan estimates of total microbial species diversity on Earth (estimated at 10^12^ (Locey and Lennon 2016)), he challenged the sequenced-based species concept (95% average nucleotide identity (Jain et al. 2018)) and argued for a more ecological concept of microbial species, where these are defined by their specific role in the local habits they occupy, at scales relevant to microbes (millimeters). He ended his lecture by speculating on what might be the physiologies and evolutionary rates of natural microbial populations (Kolter et al. 2022).

**Margaret McFall-Ngai** (Carnegie Institution for Science, Pasadena, USA) presented support for the concept that we are in an era that goes beyond the ‘New Microbiology’ to something more expansive—a comprehensive change across the field of life sciences that demands the development of a ‘New Biology’ (McFall-Ngai et al. 2013, Bosch and Hadfield 2020). This imperative emerges from changes in technology, notably next-generation sequencing (Schuster 2008), which have revealed that the microbial world is foundational to the health of all corners of the biosphere. McFall-Ngai presented support for the idea that the growing recognition and acceptance of this conceptual framework demands a full integration of the fields of micro- and macro-biology.

## Regulatory mechanisms in bacterial gene expression or behavior

**Inigo Lasa** (University of Navarra, Pamplona, Spain) focused his lecture on overlapping transcription. Overlapping transcription occurs when the same region of DNA is used to generate more than one transcript. The advancement of genome-scale protein and RNA measurement tools is continuously uncovering new examples of overlapping transcription in bacterial genomes. However, the true role of this process remains in most cases unknown. In his lecture, Lasa reviewed the types, functions, and significance of overlapping transcription in virus and bacteria (Wright et al. 2022). He then examined how overlapping transcription between 5’ and 3’ UTRs can serve to coordinate the expression of neighboring genes (Sesto et al. 2013). He ended his lecture describing the utility of a particular type of overlapping transcription, called non-contiguous operons (Sáenz-Lahoya et al. 2019) to regulate the transition from lysogenic to lytic existence in bacteriophages.

**Petra Dersch** (University of Münster, Germany) discussed RNA-based regulatory mechanisms in bacterial pathogenesis, using enteropathogenic *Yersiniae* as an example, with an emphasis on the identification of regulatory and sensory RNAs and genome-wide small RNA and RNA-binding protein discovery (Righetti et al. 2016, Nuss et al. 2017). Using different RNA-seq approaches/technologies to isolate regulatory RNAs important for virulence allowed to identify global and specific RNA regulators, that influence the expression of crucial virulence factors, such as the type III secretion system and its effectors, and the copy number of the *Yersinia* virulence plasmid in response to temperature, ions, and nutrients. The identification of similar complex integrated post-transcriptional networks in many bacterial pathogens highlights the exquisite control circuits used by pathogens to adjust and fine-tune virulence and their biological fitness during infection (Hör et al. 2018).

**Tâm Mignot** (CNRS, Marseille, France) first briefly discussed why microbial soil ecosystems have a major impact on the environment and that, as metagenomic studies are starting to unravel their overwhelming complexity, it remains difficult to assess the contribution of individual species. In all soils, predatory bacteria are found where they are hypothesized to play a major balancing function (Petters et al. 2021). He introduced *Myxococcus xanthus*, a deltaproteobacterium that moves collectively to hunt and kill entire prey communities. Because of its high genetic tractability, this organism has been a model system to study how single cells detect and kill prey, which has opened systems-level studies to understand how thousands of cells self-organize into swarming predatory packs (Seef et al. 2021). Specifically, he showed that this can be achieved thanks to the combination of high-throughput imaging combined with machine-learning and mathematical modeling (Panigrahi et al.^2021^). Such analysis uniquely reveals emergent properties of cellular groups as has been seen for example in animals, in fish schools or herd of sheep. He concluded that the future challenge will be to link these studies performed in laboratory context to the actual *Myxococcus* ecology, which will require defining the interactions that this predator encounters in its real ecological niche.

## Infection biology, antibiotic resistance, antimicrobial peptides, innate immunity, and COVID19

**Melanie Blokesch** (EPFL, Lausanne, Switzerland) summarized our current understanding of *Vibrio cholerae* biology, drawing on over 150 years of research. Sadly, cholera remains a major burden in many developing countries, where it is transmitted via contaminated water. However, volunteer challenge-studies in the 1970’s revealed that the infectious dose required to cause cholera-like symptoms is actually incredibly high (∼10^9^) (Hornick et al. 1971). This sparked several major lines of research, in particular, how *V. cholerae* survives the acidic milieu of the stomach and how it overcomes the colonization barrier exerted by the intestinal microbiota. The demonstration that *V. cholerae* adheres strongly to chitinous surfaces (Adams et al. 2019), where it produces the interbacterial killing type VI secretion system suggests a role for environmental triggers in these processes (Borgeaud et al. 2015).

**Carmen Buchrieser** (Institut Pasteur, Paris, France) described many aspects of intracellular bacteria. The aphid endosymbionts—the first known intracellular bacteria—were discovered at the beginning of the 20^th^ century by Umberto Pierantoni and further characterized by Paul Buchner. Research on intracellular pathogens then fully emerged in the mid-twentieth century and was boosted with the description of *Salmonella* growing inside cultured, epithelial cells and *Listeria monocytogenes* being released in the cytosol and then spreading from cell to cell. These findings led to the classical definition of extracellular bacteria and intracellular bacteria. Once inside a eukaryotic cell, bacteria either escape from the phagosome shortly after invasion the so-called cytosolic pathogens or they avoid phagosome-lysosome fusion and replicate in a specialized pathogen-containing vacuole, the so-called vacuolar pathogens. Breaking this divide, recent reports show that cytosolic and vacuolar lifestyles are not mutually exclusive.

To adopt these different lifestyles and to reside in different niches, intracellular bacteria have developed myriad mechanisms and strategies that allow them to exploit the eukaryotic cell as host. An excellent example is *Legionella pneumophila*, an intracellular pathogen of amoeba and human cells (Hilbi and Buchrieser 2022). This bacterium secretes proteins that mimic eukaryotic functions and modulate many signaling pathways in the protozoan as well as the human host cells (Mondino et al. 2020). Two of these proteins, as an example, target the nucleus and work together to modulate the chromatin landscape of the host. One (RomA) encodes histone methyl-transferase activity and the second one (LphD) encodes histone deacetylase activity (Rolando et al. 2013, Schator et al. 2021).

**Athanasios (Nassos) Typas** (EMBL, Heidelberg, Germany) gave a talk on antimicrobial resistance (AMR) which is emerging as one of most serious public health threats of our times. He presented our current knowledge about the size of the problem and discussed the reasons for the current crisis. The lack of new antibiotic discovery is one of them. New technological advances that empower activity-based discovery were presented, including new genetic, computational and biochemical methods to identify the mode-of-action of new compounds. At the same time, developing new antibiotics without understanding what drives AMR, and devising strategies to delay, prevent or revert resistance, is a Sisyphean effort. Nassos presented our current understanding on this topic, highlighting the risks that non-antibiotic drugs may pose for AMR development (Maier et al. 2018), the advantage that combinations and narrow-spectrum therapies may present for avoiding resistance (Brochado et al. 2018) and for decreasing the collateral damage to our gut microbiota (Maier et al. 2021).

**Bruno Lemaitre** (EPFL, Lausanne, Switzerland) discussed the role of antimicrobial peptides, which are produced by animals, plants and bacteria (Datta and Roy 2021). Despite many studies, how antimicrobial peptides contribute to host defense was unclear until only recently. The single mutation methodology that prevails today has obvious limits in the study of immune effectors, which often belong to large gene families. Leveraging a new set of single and compound antimicrobial peptide gene deletions in a controlled genetic background, the laboratory of Bruno Lemaitre has systemically analyzed the function of *Drosophila* antimicrobial peptides. Their studies confirm the importance of antimicrobial peptides notably against Gram-negative bacteria. They revealed that they can function either additively or synergistically against some microbes, but in some cases, antimicrobial peptides exhibit striking specificity, with one peptide contributing most of the resistance against a specific pathogen (Hanson et al. 2019, Carboni et al. 2022).

A number of studies in *Drosophila* and *C. elegans*, and also in vertebrates, have recently implicated antimicrobial peptides in processes as diverse as behavior, neurodegeneration and tumor clearance. Bruno discussed a model explaining how antimicrobial peptides can disrupt the membranes of aberrant host cells, that may become more negatively charged due to the exposure of phosphatidylserine (Hanson and Lemaitre 2020). He argued for the importance of studying these innate immune effector contributions to defense, and how they might impact host function.

**Andrea Ablasser**, (EPFL, Lausanne, Switzerland), gave the EMBO lecture. She shared her recent research on DNA-mediated activation of innate immunity through the cGAS-STING signaling pathway. In the first part of her talk, she showed how sensing of a cell's own DNA by cGAS can be beneficial in alerting the host to damaged cells on route to malignant transformation (Glück et al. 2017). She then went on to explain safeguard mechanisms that restrict sensing of self-DNA in normal cells, largely focusing on the role of the nucleosome in sequestering cGAS in the nucleus (Pathare et al. 2020). In the last part of her talk, she pointed out how self-DNA sensing can contribute to the pathogenesis of inflammatory conditions, and she shared unpublished work on its role in natural ageing (Decout et al. 2021).

**Rino Rappuoli** (GSK, Sienna, Italy) gave the EMBO science policy lecture. He reminded us that the rapid development of vaccines and human monoclonal antibodies during the Covid-19 pandemic were a miracle of ingenuity and modern science. Thanks to them, 20 million deaths and a huge number of hospitalizations were prevented and today we are having almost a normal life. Vaccines were developed at the unprecedented speed of 10 months thanks to the advent of fully synthetic ‘digital’ (Pizza et al. 2021) mRNA vaccines and to the huge investment from the public sector that allowed to do the vaccine development steps in parallel instead of doing them sequentially. He said that the 12.5 billion dollars that the US government invested for vaccine development were paid back just by anticipating the availability of the vaccines by 12 hours. He concluded that while present vaccines have done a great job in preventing mortality and severe disease, for the future we need also vaccines that prevent infection.

## Microbial assemblies, viromes, symbiosis, and phage defense systems

**Jean-Marc Ghigo** (Institut Pasteur, Paris, France) presented an overview of bacterial biofilms. Using specific recent examples, he presented key aspects of how biofilms are built, from specific and non-specific adhesion to complex metabolic cooperations and social interactions. He described how the physicochemical heterogeneity within the biofilm environment enables the emergence of novel properties, from the clinically problematic high tolerance to antibiotics to the expression of biofilm-specific metabolites and functions (Jo et al. 2022). While the study of the biofilm lifestyle *in vitro* and *in vivo* still raises important scientific and technical challenges, it contributed to uncover new aspects of bacterial biology (Béchon et al. 2022, Létoffé et al. 2022) and transition from classical pure culture studies to much needed multispecies microbiology.

**Karina Xavier** (Gulbenkian Institute, Lisbon, Portugal) focused her talk on bacterial interactions in gut microbial communities. Bacteria communicate through small chemical molecules to engage in group behaviors by a process called quorum sensing. It is well established that quorum sensing is crucial in dual-partner symbioses and pathogenesis. Karina Xavier showed that members of the mammalian gut microbiota also produce quorum sensing signals. Her group established a mouse model to manipulate quorum sensing in the mammalian gut and is using a combination of mathematical models, gnotobiotic mice colonized with defined microbiota communities, and transcriptomics approaches to study the role of quorum sensing in gut microbiota resilience (Thompson et al. 2015, Thompson et al. 2016). She explained how in the mammalian gut microbe-microbe interactions can affect microbiota composition and function through nutrition competition (Oliveira et al. 2020) or sharing. Recent work shows that bacteriophages can eavesdrop on bacterial communication, bringing a new layer of complexity to this fascinating microbial community (Silpe and Bassler 2019).

**Colin Hill** (University College, Cork, Ireland) made the case that the role of the virome in shaping complex bacterial population structures and in impacting bacterial functionality remains unclear (Shkoporov et al. 2022). An example was presented of the Crassvirales, the most abundant viral Order of the human gut. The Crassvirales are enormously diverse, with almost 300 individual species, and have been shown to persist in high numbers within individuals over extended periods (Shkoporov et al. 2019). How can these bacteriophages persist without eliminating their hosts? It was shown that the phage crAss001 and *Bacteroides intestinalis* are engaged in a mutually beneficial relationship at population levels, mediated by phase variation of multiple capsular polysaccharides (Shkoporov et al. 2021). This ’dance’ increases the fitness of the bacterium, while ensuring the continued existence of the phage.

**Martin Kaltenpoth** (Max-Planck Institute, Jena, Germany) summarized the current state of knowledge on microbial symbionts in insects. He emphasized how localization, transmission route, and functional contribution to the host's fitness are intimately connected and how they affect the ecology and evolution of the interacting partners. He reported on novel findings that extreme genome reduction not only occurs in intracellular bacteria, but also extends to extracellular symbionts that are intimately associated with their beetle hosts. Furthermore, his research detailed the chemical contributions of defensive symbionts to their insect hosts and provided evidence for both expansions and contractions of insects’ ecological niche space by the help of microbial symbionts (Salem et al. 2017, Reis et al. 2020, Janke et al. 2022).

**Joël Doré** (University Paris-Saclay & INRAE, Jouy-en-Josas, France) gave an update of human gut microbiome science, stressing recent findings that have re-enforced the importance of enterotypes and intestinal microbiome richness as health relevant stratifiers, including in clinical contexts such as neurological or metabolic conditions and cancer therapy where microbiome features can be predictors of risk or response to treatment (Solé et al. 2021). Joel presented new results that demonstrate the importance of host-microbiome symbiosis, its relevance for health and the associated innovation paths likely to lead to translational applications (Van de Guchte et al. 2020). These included (i) symbiosis-status monitoring by Medical Biology Labs for clinicians, (ii) preventive applications of food grade bioactives involving probiotics and/or live biotherapeutic products in combinatorial approaches, as well as (iii) full ecosystem microbiota transfer illustrated by its application in cancer therapy (Malard et al. 2021).

**Nathalie Rolhion** (INSERM, Paris, France) discussed the objectives and challenges of fecal microbiota transplantation (FMT). FMT consists of administering a preparation of faecal matter from a healthy subject to a patient suffering from a pathology linked to an alteration of the intestinal microbiota equilibrium, with the aim of increasing overall microbial diversity and restoring the microbiota's functions. She covered the history of FMT, its spectacular success in the treatment of recurrent *Clostridioides difficile* infection (van Nood et al.^2013^), some first encouraging data for other pathologies (notably inflammatory bowel diseases (Benech and Sokol 2020)), and the questions surrounding this practice, its risks, its future (Danne et al. 2021) and its potential as a mechanistic discovery tool.

**Rotem Sorek** (Weizmann Institute, Rehovot, Israel) reported on the emerging field of the bacterial pan-immune system. By systematically analyzing tens of thousands of microbial genomes for the presence of ‘defense islands,’ genomic areas where defense systems cluster, Sorek identified many dozens of novel defense systems which he later verified experimentally (Doron et al. 2018). Mechanistic biochemical analyses showed multiple new mechanisms of action, including systems that rely on second messenger signaling to employ defense (Cohen et al. 2019), systems that necessitate reverse transcription of non-coding RNAs (Millman et al. 2020), and systems that produce small-molecule compounds that inhibit phage replication (Bernheim et al. 2021). A remarkable aspect of Sorek discoveries is the understanding the many anti-viral defense mechanisms are conserved between bacteria and humans (Wein and Sorek 2022).

## Other topics: synthetic biology, physics, and X-inactivation

**David Bikard** (Institut Pasteur, Paris, France) who gave the EMBO Yip lecture, introduced the field of Synthetic Biology, presenting how the genetic engineering of microbes could contribute to the sustainable development goals. He then gave an overview of the progress made in ‘DNA writing’ technologies from the first recombinant DNA of Paul Berg to novel DNA synthesis machines and novel CRISPR techniques to modify DNA or control gene expression (Anzalone et al. 2020, Vigouroux and Bikard 2020). When adapted to high-throughput screens, these methods can generate large amount of data which can feed computational models used in the design of novel organisms, while also providing novel biological insights. David presented more specifically how CRISPR screens could be used in his laboratory to revisit the concept of gene essentiality at the level of the bacterial species (Rousset et al. 2021).

**KC Huang** (UCSF, San Francisco, USA) discussed applications of physics and modeling to complex biological systems spanning multiple length scales. First, he gave a brief introduction to scaling laws and to principles of statistical physics, and showed that entropy is sufficient to explain the translation of the *Listeria monocytogenes* protein ActA. Next, he discussed their recent findings that most pairs of gut commensals isolated from a human stool sample interact mainly through nutrient competition. They were able to use metabolomics data to build and validate a predictive consumer-resource model of community assembly. Finally, he showed that the landscape of resource consumption is a better predictor of function than taxonomy (Halladin et al. 2021, Ho et al. 2022).

**Edith Heard** (EMBL, Heidelberg, Germany) delivered the EMBO women in science lecture. She first described her own career path from studying physics in Cambridge where she shifted to biology and then to a PhD working on cancer. She was a post-doctoral fellow at the Pasteur Institute where she started to investigate X-chromosome inactivation. She was recruited by the CNRS in 1993 and moved to the Curie Institute as a junior group leader where she later became the director of the department of Genetics and Biology of development. In 2012 she was elected as professor of Epigenetics and Cellular Memory at the Collège de France. Since 2019, she has been the Director General (DG) of the European Molecular Biology Laboratory (EMBL) where she has set up a new and ambitious program entitled Molecules to Ecosystems (https://www.embl.org/about/programme/) that includes new themes such as Microbial ecosystems and Planetary Biology seeking to explore life in context. In addition, the whole organization of EMBL as highlighted in the talk is promoting women in science in several ways including mentorship programmes for young female scientists and a strategy to promote equality, diversity and inclusion at all levels. Edith Heard's lab at EMBL Heidelberg continues to work on the epigenetics paradigm of X inactivation and most recently on genes on the ‘inactivated’ X chromosome that escape X inactivation and underlie phenotypic variation as well as some sex biased pathologies (For a recent review: Loda et al. 2022)

## Conclusion

In summary, this lecture course was extremely interactive. Students showed great interest in discussing all topics with the lecturers. These discussions took place, not only at the conclusion of each lecture but also during the course, at all meals and the many other opportunities to meet in the beautiful island of Spetses.

**Figure 2.**
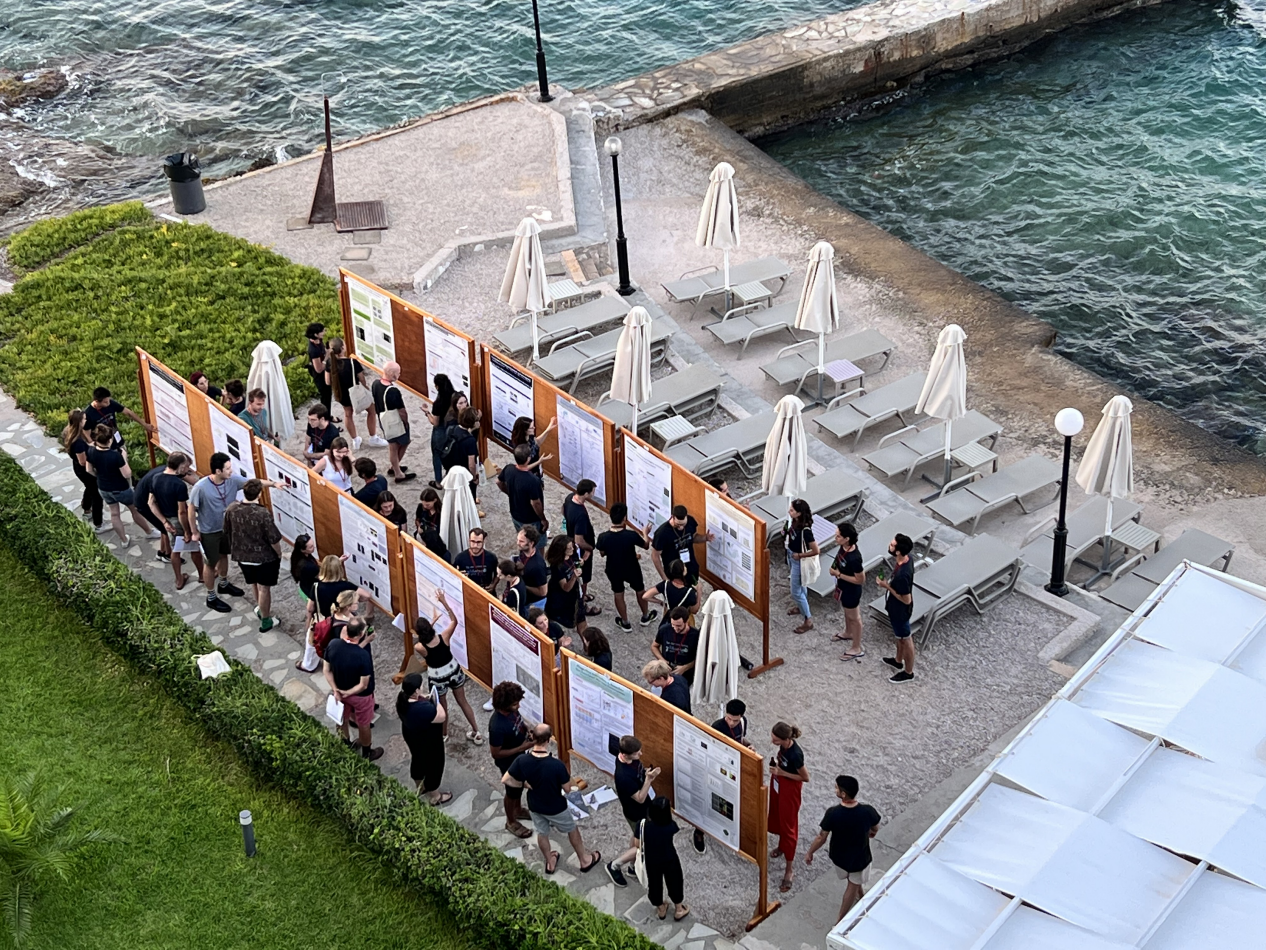
Poster session at seaside.

**The poster sessions** were particularly animated. The nice weather made it possible to have them at seaside (Fig. 2). Amazingly, the students self-organized a third and a fourth poster session to be able to learn about their friends' work. Three students were selected as winner of sponsored poster prizes and gave talks on the last day.

**Presentations of papers by students**: All students, in randomly selected groups of three, were assigned an article from one of the lecturers. They analyzed the paper with the lecturer serving as mentor and then gave oral presentations during the two last days. This meant a fair amount of extra work for the students but they all were enthusiastic about the activity as it allowed them to interact closely with the lecturer. The student presentations were all excellent.

**Figure 3.**
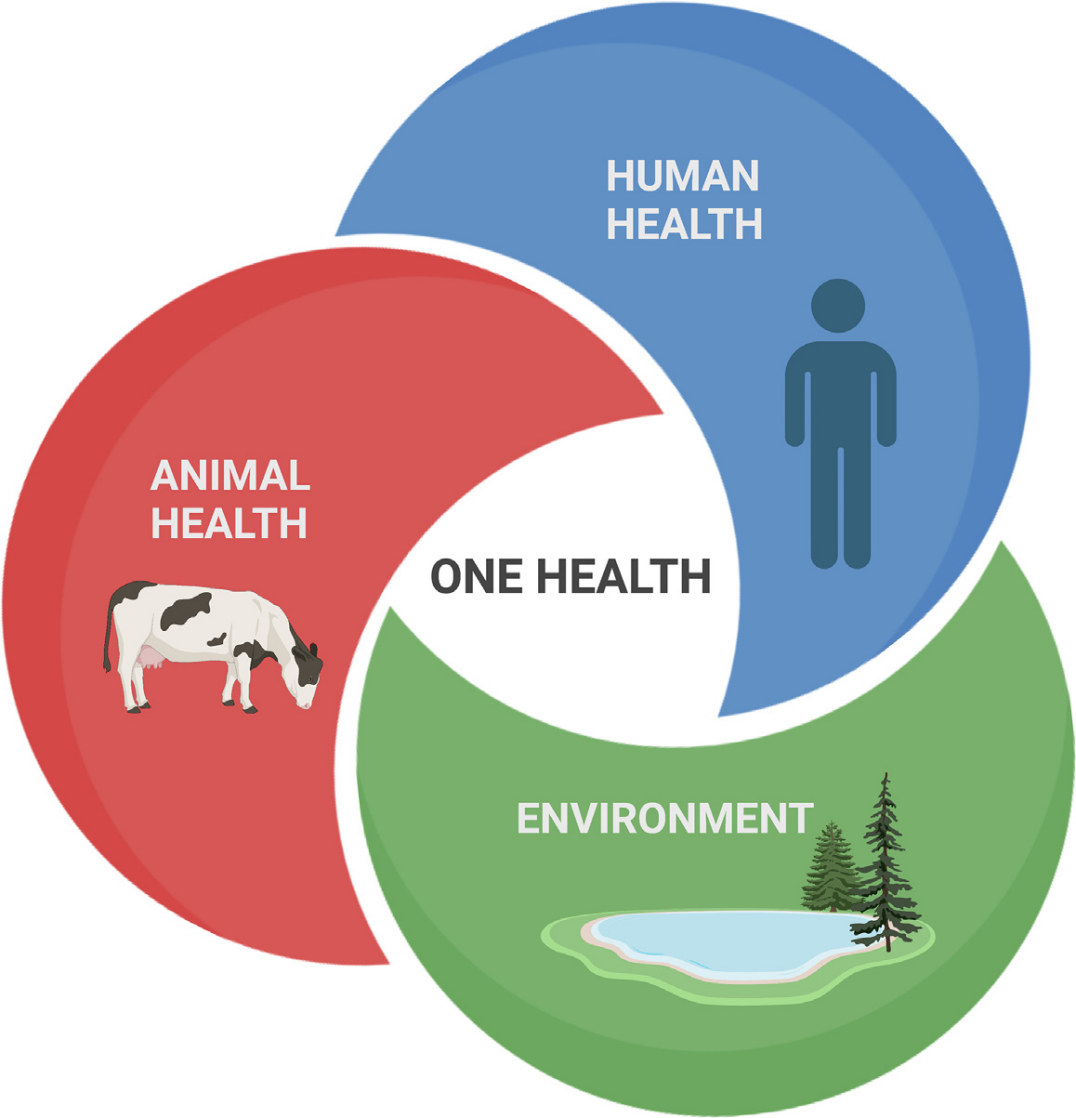
Schematic representation of the One Health concept (design by Nazgul Sakenova, EMBL-Heidelberg, student at the course).

**Two ‘trio sessions’** were organized, one entitled ‘Breakthrough advances due to novel high throughput technologies’ (Bikard, Typas, and Huang) and the second entitled ‘Emerging diseases: the concept of one health’ (Hill, Buchrieser, Kaltenpoth). Each lecturer presented the topic for five minutes and lively discussions followed. In the first trio, lecturers insisted on the fact that technologies have dramatically modified how science is performed. The second trio drove home the critical importance of the concept of one health (Fig.3) .

To conclude, it is very clear that these types of ‘full immersion’ summer courses provide unique opportunities for advanced trainees in the life sciences. Through such courses the students invariably learn of the latest developments in their discipline, get to interact closely with the faculty and establish long-lasting professional connections with their peers. Given their very special nature as an early career-building tool, every effort should be made to assure their continued success. In this regard, the outstanding record of the many successful iterations of the Spetses microbiology course provides a solid foundation for its future. With continued support from key funding sources, we are confident that the future editions of this very special course will succeed in attracting outstanding faculty and students.
